# 

**Published:** 1897-11-06

**Authors:** 


					THE HQ&PITAL. ABSOLUTELY PURE, therefore BEST. Kov. 6iu, 1897.
CADolcuofYS rf H *! CAD-""Y'S
11 The standard of highest purity at resent u?ALKALIES USED,
attainable Lancet
0
No. 580. Vol. XXIII. $*?*?} Saturday,
Nov. 6th, 1897.
[ see p?92 j PRICE TWOPENCE.

				

